# Ultrasmall Luminescent Metal Nanoparticles: Surface Engineering Strategies for Biological Targeting and Imaging

**DOI:** 10.1002/advs.202103971

**Published:** 2021-11-19

**Authors:** Xiaoxi Luo, Jinbin Liu

**Affiliations:** ^1^ Key Laboratory of Functional Molecular Engineering of Guangdong Province School of Chemistry and Chemical Engineering South China University of Technology Guangzhou 510640 China

**Keywords:** imaging, luminescent metal nanoparticles, surface engineering, biological targeting, theranostics

## Abstract

In the past decade, ultrasmall luminescent metal nanoparticles (ULMNPs, *d* < 3 nm) have achieved rapid progress in addressing many challenges in the healthcare field because of their excellent physicochemical properties and biological behaviors. With the sharp shrinking size of large plasmonic metal nanoparticles (PMNPs), the contributions from the surface characteristics increase significantly, which brings both opportunities and challenges in the application‐driven surface engineering of ULMNPs toward advanced biological applications. Here, the systematic advancements in the biological applications of ULMNPs from bioimaging to theranostics are summarized with emphasis on the versatile surface engineering strategies in the regulation of biological targeting and imaging performance. The efforts in the surface functionalization strategies of ULMNPs for enhanced disease targeting abilities are first discussed. Thereafter, self‐assembly strategies of ULMNPs for fabricating multifunctional nanostructures for multimodal imaging and nanomedicine are discussed. Further, surface engineering strategies of ratiometric ULMNPs to enhance the imaging stability to address the imaging challenges in complicated bioenvironments are summarized. Finally, the phototoxicity of ULMNPs and future perspectives are also reviewed, which are expected to provide a fundamental understanding of the physicochemical properties and biological behaviors of ULMNPs to accelerate their future clinical applications in healthcare.

## Graphical abstract

Ultrasmall luminescent metal nanoparticles (ULMNPs, *d* < 3 nm) have achieved rapid progress in the past decade in addressing many challenges in healthcare. The systematic advancements in the biological applications of ULMNPs, from bioimaging to theranostics, is provided in detail with an emphasis on the versatile surface engineering strategies for enhanced biological targeting and imaging.

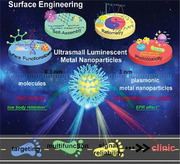

## 1 Introduction

Ultrasmall luminescent metal nanoparticles (ULMNPs, mainly including Au, ^[^ ^1^, ^2^, ^3^, ^4^, ^5^ ^]^ Ag,^[^ ^6^, ^7^, ^8^, ^9^, ^10^ ^]^ and Cu^[^ ^11^, ^12^, ^13^, ^14^, ^15^ ^]^) are typically classified as atomically precise metal nanoclusters and metal nanoparticles with core sizes below ≈3 nm. The quantum confinement effect begins to appear, and the optical and electronic properties are typically dominated by electrons in discrete energy levels, such as semiconductor quantum dots (QDs),^[^ ^16^ ^]^ which are different from the delocalized electrons observed in plasmonic metal nanoparticles (PMNPs).^[^ ^17^ ^]^ Unlike semiconductor QDs, the emission associated with ULMNPs does not appear to be purely associated with the quantum confined metal core effect, but is highly dependent on the surface state chemistry, indicating that the emission origin of ULMNPs is the result of complicated interactions between the metal core and the metal‐ligand surface.^[^ ^5^ ^]^ Because surface chemistry plays a critical role in the luminescence of ULMNPs due to the ligand‐metal charge transfer (LMCT) mechanisms, tuning the surface chemistry is expected to serve as an efficient method to tune the luminescence properties of ULMNPs.

ULMNPs with discrete energy states not only exhibits outstanding tunable optical properties,^[^ ^18^, ^19^, ^20^ ^]^ but also display unique physicochemical properties and biological behaviors,^[^ ^21^, ^22^, ^23^, ^24^, ^25^, ^26^ ^]^ which act as distinctive bridge materials between the small molecules and traditional plasmonic metal nanoparticles (PMNPs) to address many challenges in the healthcare field.^[^ ^27^, ^28^, ^29^, ^30^, ^31^ ^]^ PMNPs are often selectively transported to disease sites (e.g., tumors) with much higher concentrations and longer retention times than small molecules (e.g., fluorescent dyes) through the well‐recognized enhanced permeability and retention (EPR) effect.^[^ ^32^, ^33^, ^34^, ^35^, ^36^, ^37^ ^]^ However, unlike small molecules with fast clearance and low body retention,^[^ ^38^, ^39^, ^40^ ^]^ PMNPs often severely accumulate in the reticuloendothelial system (RES) organs (e.g., liver and spleen),^[^ ^41^, ^42^, ^43^, ^44^, ^45^ ^]^ which could lead to potential long‐term toxicity and severe systemic side effects.^[^ ^46^, ^47^ ^]^ The ULMNPs with in vivo hydrodynamic diameters (HDs) below the kidney filtration threshold (KFT, ≈5.5 nm),^[^ ^48^ ^]^ can pass through the glomerular filtration barrier and be excreted from urine with high biocompatibility,^[^ ^49^, ^50^, ^51^ ^]^ which shows the integrated advantages of both PMNPs (e.g., high targeting efficiency, high signal output, and multiple modalities) and small molecules (e.g., efficient excretion, low body retention, and high biocompatibility), holding great potential in clinical translation.

With the sharp shrinking size from PMNPs to ULMNPs, the contributions from the surface increase significantly,^[^ ^52^ ^]^ which brings both opportunities and challenges for surface engineering.^[^ ^53^, ^54^, ^55^, ^56^ ^]^ Specifically, ULMNPs with the advantages of ultrasmall size scale provide distinctive building blocks for self‐assembly into uniform nanostructures with controllable size and shape,^[^ ^57^, ^58^, ^59^ ^]^ rendering new properties and functions for advanced biological applications ranging from multimodal imaging to nanomedicine. A slight change in the surface chemistry (e.g., surface coverage, charge, and hydrophobicity) of ULMNPs can significantly affect their optical properties (e.g., absorption and emission) and biological behaviors (e.g., cellular interaction, organ distribution and excretion pathways),^[^ ^60^, ^61^, ^62^, ^63^, ^64^ ^]^ which indicates great opportunities in the application‐driven surface engineering of ULMNPs for advanced biological applications. However, some of the well‐established surface engineering strategies from PMNPs would hardly be adaptable to ULMNPs with a unique metal (0) core and metal (I)‐thiolate shell structures,^[^ ^65^, ^66^, ^67^ ^]^ which reminds chemists to further disclose the unique surface engineering strategies for ULMNPs.

**Figure 1:**
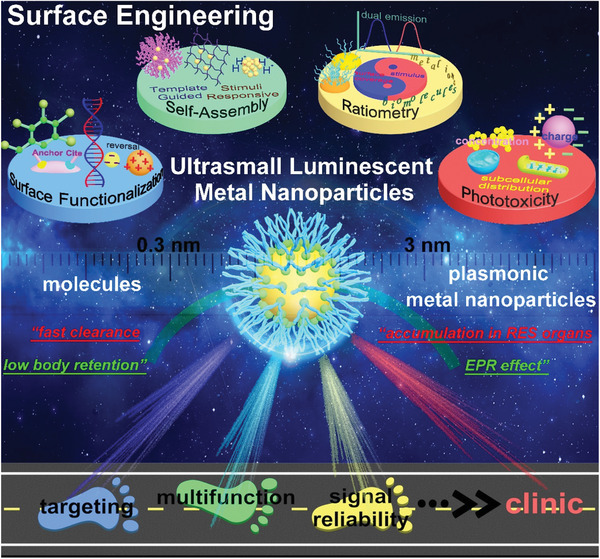
Surface engineering strategies of ultrasmall luminescent metal nanoparticles (ULMNPs) for biological targeting and imaging.

In this review, we present an overview of our efforts in the surface engineering of ULMNPs for biological targeting and imaging (**Figure** **1**), emphasizing the versatile strategies for surface engineering of ULMNPs. As the biological interactions between ULMNPs and targets are highly related to surface functionalization, the surface functionalization strategies and principles for enhanced targeting abilities are first summarized. We then systematically introduce the self‐assembly strategies of ULMNPs for fabricating multifunctional nanostructures for multimodal imaging and nanomedicine. We will also discuss surface engineering strategies of ratiometric ULMNPs to enhance the imaging stabilities and sensitivities to address the imaging challenges in complicated bioenvironments. Finally, we critically review the phototoxicity of ULMNPs and present future perspectives to accelerate their clinical applications in disease diagnosis, monitoring, and treatment.

## 2 Surface Functionalization Strategies and Principles

ULMNPs are typically considered as a type of molecule‐like optical nanoprobe with low nonspecific retention,^[^ ^50^ ^]^ high metabolic clearance,^[^ ^49^ ^]^ and fewer background tissue interactions.^[^ ^31^ ^]^ The emergence of renal‐clearable ULMNPs could potentially address some of the safety concerns of the large PMNPs with long‐term nonspecific accumulation in RES organs.^[^ ^27^ ^]^ However, the rapid renal clearance of ULMNPs raises other issues, including short blood circulation, weak EPR effect, and low targeting efficiency.^[^ ^68^ ^]^ Surface functionalization of ULMNPs is becoming crucial to further control their stability, circulation, retention, and interaction with biological environments.

**Figure 2:**
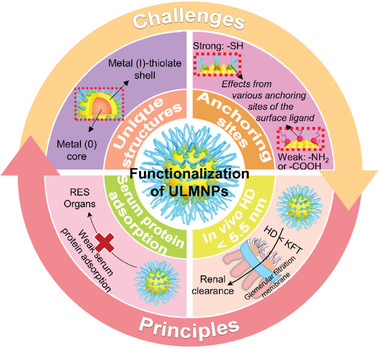
Surface functionalization challenges and principles of ULMNPs.

The surface ligand exchange strategy has typically been applied to the functionalization of PMNPs with various thiolate active‐targeting ligands (e.g., glucose,^[^ ^69^ ^]^ DNA,^[^ ^70^, ^71^ ^]^ and antibodies^[^ ^72^ ^]^) for enhanced targeting and accumulation at the tumor site. However, the functionalization of renal‐clearable ULMNPs should consider the following challenges and principles (**Figure** **2**): 1) the unique metal (0) core and metal (I)‐thiolate shell structures make it difficult to develop a well‐developed thiolate ligand exchange strategy; 2) the effects of both the strong anchoring sites (e.g., —SH) and weak anchoring sites (e.g., —COOH and —NH_2_) of the surface ligands due to the ultrasmall size; 3) maintaining in vivo HD below KFT (≈5.5 nm) for efficient renal clearance after circulation from the body; and 4) enabling the surface resistance to serum protein adsorption in biological environment to avoid fast RES sequestration.

### 2.1 Controllable Functionalization of DNA Molecules

**Figure 3:**
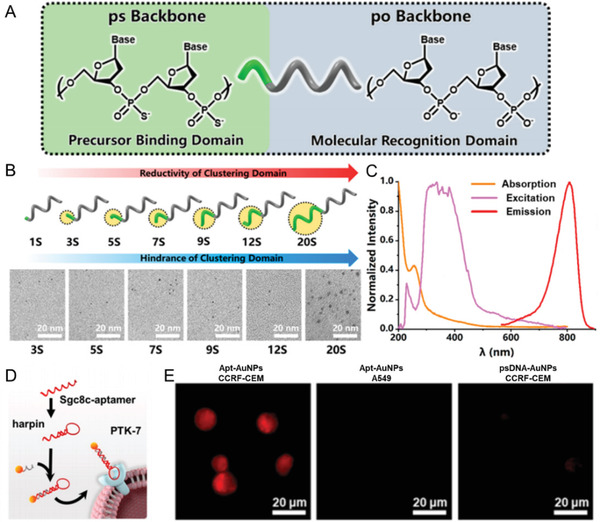
Surface functionalization of DNA molecules. A) Schematic diagram of phosphorothioate (ps)‐modified DNA (psDNA). B) Effect of ps length on the core size control of psDNA‐AuNPs (from ≈1.3 to 2.6 nm with ps length increase). C) The UV–vis and fluorescence spectra of psDNA‐AuNPs. D) Schematic diagram of the protein tyrosine kinase 7 (PTK‐7) proteins binding with the fabricated Apt‐AuNPs. E) Fluorescent imaging of Apt‐AuNPs after incubation with living cells. Reproduced with permission.^[^ ^84^ ^]^ Copyright 2020, American Chemical Society.

DNA molecules offer powerful design techniques and have unique advantages for molecular recognition.^[^ ^73^, ^74^ ^]^ Typically, DNA molecules are modified on the surface of PMNPs using alkanethiol‐capped DNA through a surface ligand exchange strategy for targeted drug delivery, disease diagnosis, and well‐organized self‐assembly. ^[^ ^75^, ^76^, ^77^, ^78^, ^79^ ^]^ However, alkanethiol‐capped DNA is difficult to modify to luminescent gold nanoparticles (AuNPs) with a unique Au(0) core and Au(I)‐thiolate shell structures using a similar ligand exchange strategy.^[^ ^1^, ^66^, ^67^ ^]^ Therefore, phosphorothioate (ps)‐modified DNA (psDNA) with programmable sulfur‐containing phosphodiester (po) backbones are ideal alternatives for increasing the affinity of luminescent AuNPs.^[^ ^80^, ^81^, ^82^, ^83^ ^]^ The rest of the domain with po backbones that show weak affinity to the gold surface act as a functional recognition domain by predictable Watson–Crick base pairing. The DNA molecules were designed with both ps domain as the template and reductant, and the po domain for molecular recognition, which were used for in situ conjugation of ultrasmall NIR‐emitting AuNPs (psDNA‐AuNPs) through a thermal reducing method (**Figure** **3**).^[^ ^84^ ^]^ The psDNA‐AuNPs showed size‐independent NIR emission (≈810 nm) with sizes that could be finely tuned from ≈1.3 to 2.6 nm through regulation of the ps length. The psDNA‐AuNPs could be controlled to bear strict DNA valence, such as one DNA (V1) and two DNAs (V2, divalent), which were good building blocks for the fabrication of well‐organized nanostructures. The psDNA‐AuNPs were then demonstrated to facilely hybridize with a sgc8c aptamer (Apt‐AuNPs) for targeting PTK7 proteins, overexpressed on the CCRF‐CEM cell membranes, which showed enhanced specific targeting abilities. Since there are many well‐established protocols in DNA technologies, this in situ synthesis strategy would provide a facile pathway to enable the ULMNPs with various new functionalities.

### 2.2 Glycoconjugation

**Figure 4:**
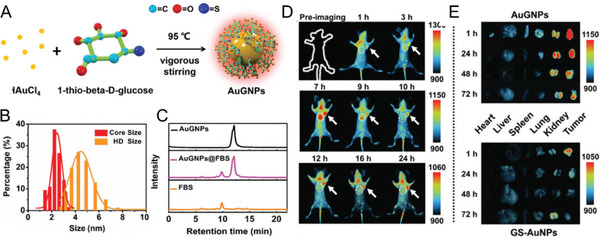
Glycoconjugation. A) Schematic diagram of the in situ synthetic process of 810 nm emitting gold glyconanoparticles (AuGNPs). B) The core‐size distributions (2.4 ± 0.4 nm) and HD (4.2 ± 0.8 nm) of the AuGNPs. C) High performance liquid chromatography (HPLC) chromatograms demonstrated that AuGNPs showed negligible serum protein absorptions (incubation conditions: 10% (v/v) fetal bovine serum (FBS) incubation for 30 min). D) Fluorescent imaging after intravenous (iv) injection of AuGNPs into MDA‐MB‐231 tumor bearing nude mice. The arrows point to the tumor area. E) Comparison of tumor targeting efficiency between AuGNPs and control group GS‐AuNPs at different post‐injection (p.i.) time points. Reproduced with permission.^[^ ^94^ ^]^ Copyright 2019, Royal Society of Chemistry.

Glycoconjugation is a promising strategy for the functionalization of nanoparticles with enhanced tumor‐targeting efficiency.^[^ ^85^, ^86^, ^87^ ^]^ Cancer cells overexpress glucose transporters and hexokinase that can recognize glucose‐functionalized nanoparticles, which could increase the cellular uptake efficiency and alter the biodistributions.^[^ ^88^, ^89^, ^90^, ^91^, ^92^, ^93^ ^]^ Meanwhile, 1‐thio‐*β*‐D‐glucose (TG) is a derivative of glucose, in which the hydroxyl group at the C‐1 position is replaced with a thiol group, which enhances the binding capability toward the metal surface. Using TG as both the surface ligand and reducing agent, ultrasmall 810 nm emitting gold glyconanoparticles (AuGNPs, ≈2.4 nm) were successfully synthesized (**Figure** **4**), ^[^ ^94^ ^]^ which showed resistance to serum protein adsorption and maintained HD of 4.2 ± 0.8 nm below KFT. The ultrasmall NIR‐emitting AuGNPs entered the cancer cells through glucose transporters, and showed not only long blood circulation with enhanced tumor‐targeting efficiency but also low accumulations in RES organs with efficient clearance. After comparison with those of the ultrasmall nonglycoparticles, glycoconjugation of ultrasmall AuGNPs led to ≈10 times and ≈2.5 times greater increase in the efficiency of cancer cell uptake and in vivo tumor targeting, respectively. The tumor targeting efficiencies of ultrasmall AuGNPs (≈2.4 nm) were higher than those of the renal‐clearable nonglycoconjugated GS‐AuNPs (≈2.5 nm) or the nonrenal‐clearable glycoconjugated large plasmonic AuGNPs (p‐AuGNPs, ≈13 nm). The findings will provide guidance in the future design of clinically translatable nanomedicines with both high tumor‐targeting efficiency and efficient clearance for the advanced diagnosis and treatment of cancer, including metastatic tumors and brain tumors with blood–brain barrier crossing capabilities.

### 2.3 Surface Functionalization with Controllable HD and Surface Charge

**Figure 5:**
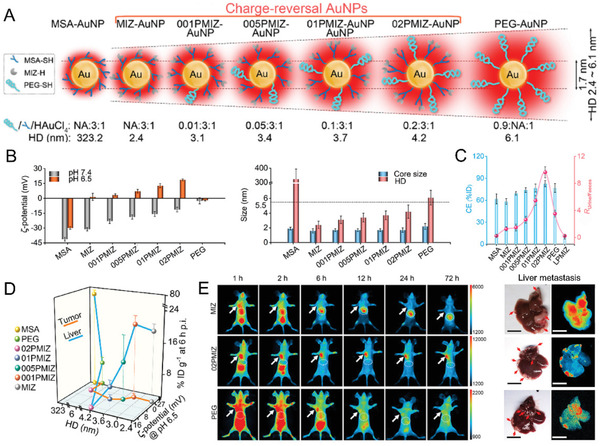
Surface functionalization of controllable surface charges and HDs. A) Schematic diagram of the 800 nm emitting AuNPs with the similar core size but different HDs and charges. NA, not available. B) Precise regulation of *ζ*‐potential values and HDs of the AuNPs. C) Total clearance efficiency (CE) calculated from both urine and feces, and the ratio values between urine and feces (*R*_urine/feces_) of the AuNPs within 168 h p.i. D) Relationships among the biodistributions in liver and tumor (at 6 h p.i.), HDs and *ζ*‐potential values of AuNPs. E) In vivo fluorescent images of the mouse iv injection with different AuNPs at different times points of p.i. (left) and ex vivo liver tissue fluorescent images of the liver‐metastasis (at 6 h p.i.). The circle and arrow indicate the liver region and the tumor location, respectively. Reproduced with permission.^[^ ^100^ ^]^ Copyright 2020, American Chemical Society.

The acidic tumor microenvironment (TME, pH 6.5–7.2),^[^ ^95^ ^]^ resulting from the increased metabolism and activated anaerobic glycolysis of cancer cells to produce acidic metabolites (e.g., lactate), has been well recognized as a promising target for tumor‐specific theranostics.^[^ ^96^, ^97^ ^]^ Since surface chemistry (e.g., HD and surface charge) determines the nanoparticle biodistributions among the tumor site and MPS organs,^[^ ^98^, ^99^ ^]^ the synergistic integration of the strategies of precise HD and surface charge regulation for the ULMNPs is critical for targeting the acidic TME. The charge‐reversal NIR‐emitting AuNPs were designed through tunable surface PEGylation to precisely tune the HD and surface charge, as well as conjugation of pH‐responsive imidazole groups to further control the charge‐reversal of AuNPs at different pH values.^[^ ^100^ ^]^ Using the ultrasmall 810 nm emitting AuNPs (core size ≈1.7 nm) with precise HDs (2.4–4.2 nm), *ζ*‐potential values (−31.2 to −11.4 mV at pH 7.4) and comparable negative‐to‐positive (*ζ*_pH 6.5_ – *ζ*_pH 7.4_, ≈28.8 mV) charge‐reversal capabilities as model, it was observed that the ultrasmall charge‐reversal AuNPs with high *ζ*‐potential values and large HD show the high tumor‐targeting specificity (**Figure** **5**). The optimized ultrasmall charge‐reversal AuNPs (HD: 4.2 nm; *ζ*‐potential: −11.4 mV at pH 7.4) showed high tumor‐targeting efficiencies (≈9% ID g^−1^) and extremely low accumulation in MPS organs (e.g., liver ≈2% ID g^−1^), resulting in high signal‐to‐noise (*S*/*N*) ratio values for the identification of cancer metastases. The small metastatic tumors (≈1 mm) in the liver were well distinguished noninvasively with high *S*/*N* ratios (≈4.6), fast response (10 min), and long imaging window time (> 6 h). In addition to the liver cancer metastasis, the tumors in other MPS organs, such as the spleen and lungs were also well imaged with *S*/*N* ratios of ≈5.2 and ≈4.5, respectively. Therefore, the charge‐reversal ULMNPs through precise HD and charge regulation would provide a facile pathway to address the long‐standing challenge in optical imaging of metastatic tumors with metal nanoparticles, which holds great promise for metastatic cancer diagnosis in future clinical practice.

### 2.4 Weak Anchor Site Effects of the Surface Ligands

**Figure 6:**
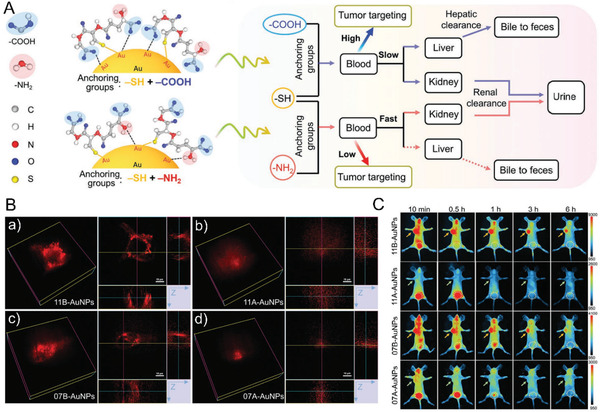
Effects of the weak anchor sites. A) Flow diagram of in vivo behaviors of the AuNPs with different anchoring sites. B) 3D‐fluorescent images of the cells incubated with 11B‐AuNPs a), 11A‐AuNPs b), 07B‐AuNPs c), and 07A‐AuNPs d) for 3 h. Scale bar, 10 µm. C) In vivo fluorescent images of tumor‐bearing mice at different p.i. time points. The arrow represents the location of tumor, and the circle indicates the area of bladder. The 11B‐AuNPs and 07B‐AuNPs with more anchoring sites of —COOH were synthesized at pH 10.0 with molar ratio of GSH to HAuCl_4_ (*R*_GSH/HAuCl4_) at 1.1 and 0.7, respectively. The 11A‐AuNPs and 07A‐AuNPs with more anchoring sites of —NH_2_ were synthesized at the corresponding conditions except at pH 2.0. Reproduced with permission.^[^ ^110^ ^]^ Copyright 2021, Wiley‐VCH.

Functionalization of nanoparticles with surface ligands is considered the most common strategy for controlling the stability, circulation, toxicity, and biological interactions. ^[^ ^101^, ^102^, ^103^ ^]^ Various surface ligands, such as peptides, proteins, and nucleic acids, can be conjugated to the Au surface via anchoring groups, such as —SH, —NH_2_, and —COOH.^[^ ^104^, ^105^, ^106^, ^107^ ^]^ The binding between —SH and Au has been long‐term recognized because of the strong covalent interaction of Au–S (≈40 kcal mol^−1^), in contrast to the relatively weak interaction of Au–O_–COOH_ (≈2 kcal mol^−1^) or Au—N_–NH2_ (≈8 kcal mol^−1^).^[^ ^108^, ^109^ ^]^ Therefore, the effects of the weak anchoring groups of —NH_2_ and —COOH are ignored during surface functionalization. Using a typical thiolate peptide, GSH with anchoring groups of —SH, —COOH, and —NH_2_, as an example, GSH‐functionalized NIR emitting AuNPs with controllable anchoring sites were synthesized in situ through pH regulation (**Figure** **6**).^[^ ^110^ ^]^ In addition to the formation of strong S–Au binding on the surface of AuNPs, the weak anchoring groups (—NH_3_^+^ and —COOH) protonated at a low pH value (e.g., pH 2.0) would facilitate the formation of N–Au anchoring sites with more exposed surface —COOH, while deprotonation at a high pH value (e.g., pH 10.0) would enhance the formation of anchoring sites of COO–Au and more exposed surface —NH_2_. The different weak anchoring sites of the AuNPs affected their behaviors significantly at both in vitro and in vivo levels: the more anchoring sites of COO–Au and more exposed surface —NH_2_ would increase cellular interactions and prolong blood circulation and retention, which would also affect the elimination pathways and increase the tumor‐targeting efficiencies. Because the typical surface ligands for the functionalization of ULMNPs contain multiple anchoring groups, the findings provide guidance for chemists to be aware of the significance of weak anchoring sites in the functionalization of ULMNPs toward future clinical translations.

## 3 Self‐Assembly Pathways for Multifunctional Nanostructures

Self‐assembly of ULMNPs into ordered and well‐defined nanoarchitectures with controllable sizes and shapes enable new properties and applications, ^[^ ^111^, ^112^, ^113^, ^114^ ^]^ which provides new opportunities to construct smart multifunctional nanostructures. Various nanoparticles have been used as different building blocks via self‐assembly.^[^ ^115^, ^116^, ^117^, ^118^, ^119^ ^]^ For PMNPs, the localized surface plasmon resonances (SPRs) of adjacent nanoparticles are coupled after assembly in close contact,^[^ ^120^ ^]^ resulting in the increase of the electric field in the gap and magnification of optical absorption, which generates various multifunctional biomedical applications (e.g., photothermal therapy, multimode imaging, and gene/drug delivery).^[^ ^121^, ^122^ ^]^ The emergence of ULMNPs has overcome some of the challenges (e.g., instability, uncontrolled aggregation, and polydispersity) faced in the self‐assembly of PMNPs.^[^ ^123^, ^124^ ^]^ With their enhanced stability and diversified surface functionalities, ULMNPs offer an excellent platform for developing multifunctional nanostructures through both template‐guided self‐assembly (e.g., polymers and peptides) and stimuli‐responsive self‐assembly (e.g., pH).

### 3.1 Template‐Guided Nanoassemblies

**Figure 7:**
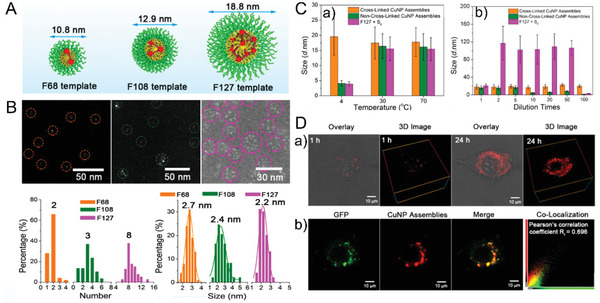
Template‐guided Cu nanoassemblies. A) Schematic illustration of the controllable encapsulated CuNP number in one Cu nanoassembly using different block copolymers as templates. B) High‐angle annular dark field (HAADF)‐Scanning transmission electron microscopy (STEM) images (up), and the analysis of encapsulated quantity and size of the CuNP assemblies. C) Excellent stability of the crosslinked CuNP assemblies at low temperature a) and with dilutions b). D) Overlay of bright and fluorescence field, and 3D images of the CuNP assemblies at different cellular incubation time points a). b) Colocalization analysis between green fluorescent protein (GFP)‐labeled lysosomes and the CuNP assemblies. Reproduced with permission.^[^ ^133^ ^]^ Copyright 2019, American Chemical Society.

Amphiphilic block copolymers (ABCs) can be used as powerful templates to self‐assemble both small molecules (e.g., doxorubicin and paclitaxel) and nanoparticles (e.g., AuNPs) into core–shell architectures with distinct sizes and shapes.^[^ ^125^, ^126^ ^]^ The small molecules or nanoparticles were loaded inside the inner hydrophobic core with the hydrophilic corona outer of the nanoassemblies, which was beneficial for prolonging circulation in blood for enhanced disease‐targeting efficiency.^[^ ^127^, ^128^, ^129^, ^130^, ^131^, ^132^ ^]^ By taking advantage of the controllable size and shape of the ABC template (e.g., Pluronic F127), a facile strategy was developed for the in situ fabrication of highly luminescent Cu nanoassemblies (**Figure** **7**).^[^ ^133^ ^]^ Owing to the crosslinking between the rigidity multidentate thiol ligand and ultrasmall CuNPs (≈2.2–2.7 nm), the absolute quantum yield (QY) of the CuNP assemblies was 7.3%, which was higher than most of the reported ultrasmall CuNPs.^[^ ^134^ ^]^ Both the size of the CuNP assemblies (e.g., ≈10.8, 12.9, and 18.8 nm) and the dominant number (e.g., ≈2, 3, and 8) of encapsulated CuNPs in an assembly could be regulated by varying the block segments (e.g., F68, F108, and F127). The CuNP assemblies were well covered by hydrophilic PEG chains on the surface, enabling the CuNP assemblies with good water solubility, biocompatibility, and high photophysical and structural stability in the harsh lysosomal acidic microenvironment (e.g., pH 4.5–6), which holds great promise for biological applications. The ABC template‐guided assembly for in situ fabrication of more highly controlled and stable CuNP‐based assemblies would stimulate more advanced downstream applications of unstable metal nanoparticles.

**Figure 8:**
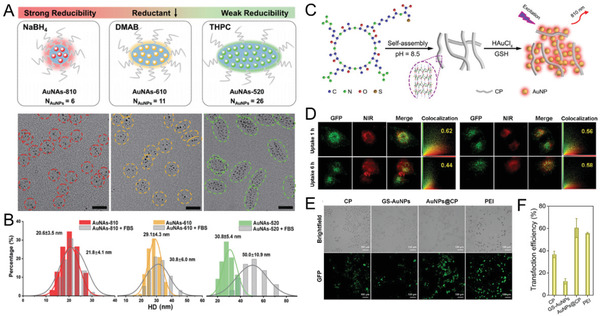
Template‐guided Au nanoassemblies. A) Controllable shape and loading of the AuNAs with different reducing agents. B) HD shows different interactions between proteins and three kinds of nanoassemblies. Reproduced with permission.^[^ ^135^ ^]^ Copyright 2020, Springer Nature. C) Schematic illustration of in situ self‐assembly process of AuNPs@CP. D) Colocalization analysis between AuNPs (red channel) (AuNPs@CP, left; GS‐AuNPs, right) and GFP‐labeled lysosomes (green channel). Pearson's correlation coefficients were shown in yellow. The reduced Pearson's correlation coefficient of AuNPs@CP between 1 and 6 h represents the effective lysosome escape process. E) Cell images after pcDNA3.1(+)‐IRES‐GFP‐p53 plasmid transfection with different vectors. F) Transfection efficiencies. Reproduced with permission.^[^ ^138^ ^]^ Copyright 2020, Springer Nature.

In addition to the enhancements in stability and QY, tunable self‐assembled shapes from spherical to elongated nanostructures could be achieved through growth regulation of AuNPs with amphiphilic block copolymers (**Figure** **8**A,B), pluronic F127, as a template.^[^ ^135^ ^]^ By tuning the reducing agents in the growth of luminescent AuNPs (≈1.7 nm), the use of different reducing agents not only changed the emission spectra of the encapsulated AuNPs, but also adjusted the hydrophobic/hydrophilic environment of the amphiphilic block copolymers to control the assembled nanostructures and the number of encapsulated AuNPs (NAuNPs). The morphologies of Au nanoassemblies (AuNAs) could be well‐controlled from spherical to elongated nanostructures with different NAuNPs (≈6, 11, and 26), which showed emission wavelengths of 810 nm (AuNAs‐810), 610 nm (AuNAs‐610), and 520 nm (AuNAs‐520), respectively. In the self‐assembly of PMNPs, the surface plasmon absorption of the formed nanostructures could red‐shift even to the NIR region depending on the interparticle distances and assembled morphology.^[^ ^136^ ^]^ However, the emission wavelengths of the nanoassemblies are governed by the different amounts of Au(I) species resulting from the different reducing abilities. The Au(I) species of the AuNAs were 40.81, 36.53, and 34.35% for AuNAs‐520, AuNAs‐610, and AuNAs‐810, respectively, further confirming that the valence states of the Au atoms affected the emission properties of the AuNAs, and the emission peak blue‐shifted upon increasing the Au(I) species. The further evaluation of their biological behaviors showed that a spherical nanostructure with a more hydrophilic surface and low values of NAuNPs resulted in prolonged blood circulation and enhanced transport of AuNPs into the tumor site as high as ≈25.3% ID g^−1^, which was much higher than most of the previously reported nanoparticles, such as PEGylated 2.5 nm AuNPs (8% ID g^−1^)^[^ ^68^ ^]^ and upconversion NPs (13.6% ID g^−1^).^[^ ^137^ ^]^ In contrast, an elongated nanostructure with a more hydrophobic surface and high values of NAuNPs resulted in short blood circulation and low tumor‐targeting efficiency, which were different from the ABC micelles for transport of small molecular drugs (e.g., paclitaxel).^[^ ^125^, ^126^ ^]^ The observation that the morphologies of the AuNAs played a significant role in governing the in vivo transport behaviors are expected to offer essential insights into the further design of biocompatible nanoassemblies as efficient nanocarriers/nanovectors for future clinical translation.

Owing to their multifunctionality, biocompatibility, and biodegradability, synthetic peptides are also perfect templates for the self‐assembly of nanoparticles into biological systems for regenerative medicine, drug delivery, and cancer therapy.^[^ ^139^, ^140^, ^141^ ^]^ The biodegradation of peptide‐guided assemblies is of great importance in achieving accurate and body‐clearable smart multifunctional nanostructures, and further reducing several concerns regarding biosafety problems.^[^ ^142^, ^143^, ^144^, ^145^ ^]^ A well‐designed cyclopeptide (CP) containing three arginines and four alanines in a cyclic ring and one cysteine on the terminal of the branched chain, could spontaneously form controllable nanofibers, which were utilized as templates for the in situ assembly of NIR‐emitting AuNPs into well‐controlled 1D nanostructures (AuNPs@CP) (Figure 8C–F).^[^ ^138^ ^]^ The self‐assembled AuNPs@CP exhibited a high emission peak at 810 nm with a QY of more than 5.9%. The AuNPs@CP were also revealed to show unique capability of rapid cellular interaction, efficient lysosome escape, as well as fast cellular efflux as compared to the unassembled ones, which resulted in high gene transfection efficiencies in the construction of HeLa cell line with ≈7.5‐fold overexpression of p53 protein. In addition, the unique 1D AuNPs@CP nanostructures were demonstrated to be robust and efficient in vivo Nanovectors, showing high diffusibility after intratumoral injection and could be eliminated through both renal and hepatic metabolic pathways with extremely low nonspecific accumulation in healthy organs. This CP‐templated in situ self‐assembly strategy offers a facile and feasible pathway to generate highly luminescent ULMNP‐based 1D nanostructures with multifunctionalities for both optical tracking and delivery.

### 3.2 Stimuli‐Responsive Functional Nanoassemblies

**Figure 9:**
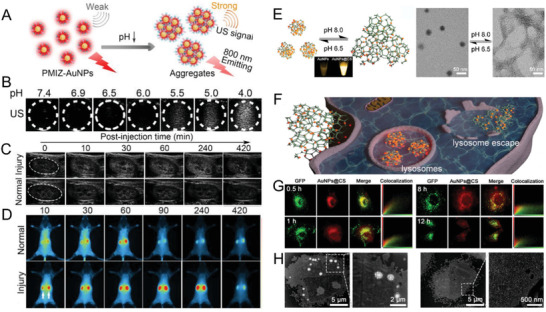
Stimuli‐responsive functional nanoassemblies. A) Scheme of pH‐induced aggregation and ultrasound contrast. B) Ultrasound imaging of PMIZ‐AuNPs at different pH values. Ultrasound imaging C) and fluorescence imaging D) of kidney injury with PMIZ‐AuNPs. Reproduced with permission.^[^ ^152^ ^]^ Copyright 2021, Wiley‐VCH. E) Self‐assembled AuNPs@CS at different pH values. The inset photo shows obvious fluorescence enhancement after assembly. F) Schematic diagram of the endocytosis and lysosome escape of AuNPs@CS. G) Subcellular distribution of AuNPs at different time points. Representative microscopic cellular images after incubation of AuNPs@CS (red) and overlay with GFP‐labeled lysosomes (green) with the colocalization analysis. H) Dark‐field (DF)‐STEM images of cells incubated with AuNPs@CS (20.0 × 10^−9^ m) for 1 h (left) and 8 h (right). Reproduced with permission.^[^ ^153^ ^]^ Copyright 2019, American Chemical Society.

Among the environmental stimuli (e.g., pH, redox, and hypoxia),^[^ ^146^ ^]^ the pH gradients have been widely applied to design smart responsive nanoassemblies. To design pH‐responsive functional nanoassemblies that could change their structures and properties under the stimuli of pH gradients, the typical strategy is the construction of reversible protonation/deprotonation systems with pH‐responsive moieties (e.g., imidazole, polyhistidine, and polyamines).^[^ ^147^, ^148^, ^149^, ^150^, ^151^ ^]^ By co‐coating with pH‐responsive zwitterionic imidazole groups, the renal‐clearable 800 nm emitting PEGylated AuNPs (PMIZ‐AuNPs, ≈1.9 nm) showed both charge‐reversal (*ζ*‐potential values: −10.9 ± 1.0 mV at pH 7.4; 17.4 ± 1.6 mV at pH 5.5) and self‐assembly (HDs: ≈3.5 nm at pH 7.4; ≈1048.7 nm at pH 5.5) capabilities toward acidic pH microenvironments, resulting in the enhanced ultrasound intensities at low pH values (**Figure** **9**A–D).^[^ ^152^ ^]^ The ultrasound signal of the pH‐responsive renal‐clearable luminescent AuNPs was demonstrated for both noninvasive fluorescence and ultrasound imaging of early kidney injury. The enhancement from both pH‐induced tubular reabsorption and in situ self‐assembly of ultrasmall luminescent AuNPs in tubular cells of the injured kidney showed enhanced ultrasound signals for noninvasive imaging of kidney injury. The discovery of tubular reabsorption of self‐assembled ULMNPs in acidic kidneys provided uniqueness in synergistic fluorescence and ultrasound imaging for future early diagnosis and treatment of renal diseases.

The integration of a sensitive emission response with a highly pH‐responsive charge conversion system would also enable self‐assembled luminescent AuNPs with more applications for both intracellular delivery and optical imaging. Using a conventional cationic polymer chitosan (CS) with isoelectric point of 6.5 as a template, pH‐responsive self‐assembled luminescent AuNPs with reversible pH‐dependent swelling and compacting structures at physiological pH range (pH 6.5–7.4) were constructed (Figure 9E–H).^[^ ^153^ ^]^ The ultrasmall AuNPs formed compacted nanostructures (≈23.5 nm) with QY increasing from 3.6% to 9.4% at low pH values (e.g., pH < 6.5). However, the compacted nanostructures could transform to swelled nanostructures with weak emissions reversibly at high pH values (e.g., pH 7.4), with a fast and sharp emission transition from pH 7.5 to 6.5. The pH‐responsive self‐assembly of the luminescent AuNPs not only increased the emission properties (>4‐fold) but also changed the surface charge and assembled size for enhanced cellular internalization (≈15‐fold). The self‐assembled AuNPs@CS showed not only strong capability for endosomal escape but also a drastic fluorescence response from an acidic lysosome to a neutral cytoplasm, which provided a feasible pathway for intracellular delivery and optical visualization of the intracellular tracking pathway.

## 4 Construction Strategies of Ratiometric ULMNPs

**Figure 10:**
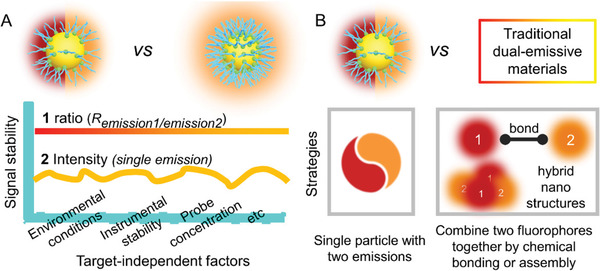
Features of dual‐emissive ULMNPs. A) Comparison of dual‐emissive ULMNPs and single‐emission ULMNPs. Dual‐emissive ULMNPs can convert fluorescence intensity signals that are susceptible to environmental interferences (e.g., environmental conditions, instrumental stability, and probe concentration) into more stable and reliable ratio signals. B) Comparison of dual‐emissive ULMNPs and traditional dual‐emissive materials. The construction strategy of dual‐emissive ULMNPs is more convenient than that of the traditional dual‐emissive materials.

The most reported ULMNPs rely on the change in the emission intensity as a recognition signal, which would be easily interfered by target‐independent factors (e.g., photobleaching, instrumental stability, environmental conditions, and probe concentration) and might cause inaccurate sensing and imaging results.^[^ ^154^, ^155^ ^]^ In quantitative imaging, dual‐emissive probes with ratiometric ability are more attractive than single‐emissive ones because of the built‐in calibration feature from the two different emissive wavelengths to avoid irrelevant influence factors (**Figure** **10**).^[^ ^156^, ^157^, ^158^, ^159^, ^160^ ^]^ There are two common design strategies for achieving ratiometric fluorescent probes based on fluorescence resonance energy transfer (FRET), intermolecular charge transfer (ICT) or excited‐state intramolecular proton transfer (ESIPT) principles: one is to introduce a second fluorophore to a fluorescent molecule/nanoparticle,^[^ ^161^ ^]^ and then utilize their separate responses as a signal response group/reference; the other is to embed two different fluorescent molecules/nanoparticles together to form a hybrid nanostructure that enables ratiometry.^[^ ^162^, ^163^, ^164^ ^]^ However, these construction strategies often involve many sophisticated coupling/chemical modification processes, and even tedious multistep synthesis/purifications.^[^ ^165^ ^]^ An ideal ratiometric ULMNP is that a single nanoparticle can intrinsically emit dual emissions without the conjugation of an additional fluorophore. Therefore, the design of intrinsic dual‐emissive metal nanoprobes is of great importance for ratiometric imaging in complicated bioenvironments.

### 4.1 Surface Coverage‐Regulated Dual‐Emissive ULMNPs

With the sharp shrinking in size, the contributions of the surface would significantly increase towards the optical and physicochemical properties of the nanoparticles.^[^ ^166^, ^167^, ^168^, ^169^, ^170^, ^171^ ^]^ For instance, with an increase in the density, rigidity, or electron‐donating capability of the surface ligands, ultrasmall AuNPs showed a large enhancement in the emission QYs.^[19,^ ^172^, ^173^, ^174^ ^]^ Through a systematic investigation of GSH‐coated AuNPs (GS‐AuNPs) emitting at 600 and 810 nm with identical sizes of ≈2.5 nm, it was discovered that the different emission wavelengths were independent of the size of the AuNPs but highly dependent on the surface coverage, with a high surface coverage resulting in strong Au(I)‐ligand charge transfer and 600 nm emission, whereas a low surface coverage led to weak charge transfer and 810 nm emission.^[^ ^60^ ^]^ Inspired by the surface coverage governed size‐independent emissions, the dual‐emissive GS‐AuNPs with emissions at both 600 and 810 nm were then synthesized through strictly controlled surface coverage by fine‐tuning the GSH/HAuCl_4_ ratio in a synthetic process. The integration of both 600 and 810 nm emission centers in a single AuNP was then discovered to cause a synergistic effect: the dual‐emissive GS‐AuNPs were sensitive to pH changes in a ratiometric manner, which was attributed to the strong energy transfer between the two emission centers in the same ultrasmall particle (2.5 nm). Single 810 nm emissive GS‐AuNPs showed negligible emission response towards pH changes, whereas single 600 nm emissive GS‐AuNPs exhibited a slight increase during pH changes from 5.0 to 9.0. In addition, the mixture of single 810 nm emissive AuNPs and single 600 nm emissive AuNPs showed independent emission responses to pH changes, significantly different from the results observed from dual‐emissive GS‐AuNPs. These differences demonstrate that the dual emissions of dual‐emissive GS‐AuNPs are from one single ultrasmall nanoparticle with two coupled emission centers that show strong energy transfer. The above findings open the possibility for the design of ultrasmall ratiometric pH nanoindicators by controlling the surface coverage of the ULMNPs.

**Figure 11:**
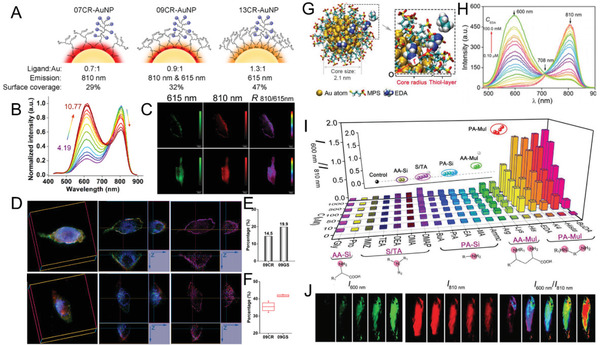
Ratiometric ULMNPs. A) CR‐AuNPs with different surface coverage and emissions. The CR‐AuNPs shows dual‐emission at the ratio of ligand to Au of 0.9. B) The pH‐dependent fluorescent spectra of dual‐emitting 09CR‐AuNPs at different pH values. The fluorescence between 615 and 810 nm shows different pH responses. C) Fluorescent images at different emission channels of living cells after treatment of 09CR‐AuNPs. D) Fluorescent and ratiometric 3D images of living cells after incubation with 09CR‐AuNPs (up) and 09GS‐AuNPs (bottom). E) The percentages of statistical ratio values >3 in ratiometric 3D images. F) Colocalization percentages of AuNPs and Lysotracker Green. Red channel: 810 nm. Green channel: 615 nm. Blue channel: Lysotracker Green. Reproduced with permission.^[^ ^175^ ^]^ Copyright 2019, American Chemical Society. G) A snap shot of an MPS‐AuNP with a few ethylenediamine (EDA) molecules in molecular dynamics (MD) simulations. Sticks show MPS, and yellow balls show Au atoms. Blue (N), cyan (C), and white (H) balls represent EDA. H) Luminescence spectra of MPS‐AuNPs with different EDA concentrations. I) Ratio values of MPS‐AuNPs after interaction with different amines. Insert showed statistical ratio values of MPS‐AuNPs upon adding of different amines (1.0 × 10^−3^ m). J) Luminescent and ratiometric images of glass catfish wrapped with MPS‐AuNPs under 37 °C at different storage time. Reproduced with permission.^[^ ^176^ ^]^ Copyright 2019, Wiley‐VCH.

The dual‐emissive AuNPs showed unique advantages in subcellular imaging and tracking (**Figure** **11**A–F).^[^ ^175^ ^]^ To enhance the cellular interaction, the dual‐emissive AuNPs were designed by co‐introduction of GSH and CR_8_ (CR‐AuNPs), a cell‐penetrating peptide that has both a thiol for binding AuNPs and a cell‐penetrating region across the plasma membrane. The CR‐AuNPs with dual emissions at both 810 and 615 nm served as powerful fluorescent probes for imaging their cellular interactions. Distinct from the single‐emitting CR‐AuNPs (810 or 615 nm) that showed lower pH dependency, the emission intensity at 810 nm of the dual‐emitting CR‐AuNPs increase while the intensity at 615 nm decreased with extracellular pH values from 7.4 to 5.8. In addition, the pH‐dependent differences in the intensity at 810 and 615 nm could be clearly displayed by the ratiometric image of the ratio value of *I*_615 nm/I810 nm_ (*R*_615/810 nm_), which demonstrates the great potential of pH‐responsive dual‐emitting CR‐AuNPs as ratiometric nanoprobes for fluorescent pH imaging of living cells. In addition, the pH‐responsive dual emissions of the CR‐AuNPs could be applied for ratiometric imaging to indicate the subcellular location and endocytosis pathways of the nanoprobe. Due to the significant acidity of lysosomes (e.g., pH 4.5–5.5), the high *R*_810/615 nm_ values (>3) in the ratiometric images could indicate the lysosome during endocytosis. Compared with the results of GS‐AuNPs, the lower percentage (*R*_810/615 nm_ > 3) was attributed to the capabilities of CR‐AuNPs in a direct cytomembrane cross without undergoing endocytosis. Therefore, the surface‐coverage‐regulated dual‐emissive AuNPs could serve as powerful self‐calibration ultrasmall nanoindicators for the investigation of cellular pH values, subcellular distributions, and cellular interaction mechanisms.

### 4.2 Stimulus‐Responsive Regulation for Ratiometric Imaging

In addition to the intrinsic emissions governed by the surface coverage that are regulated by tuning the molar ratios between surface ligands and metal precursors during the synthesis,^[^ ^60^, ^175^ ^]^ the emission of pre‐formed ULMNPs is also highly sensitive to surface coverage change induced through introduction of second ligand. For example, after introduction of tetraoctylammonium (TOA) cations to the surface of Au_22_(SG)_18,_ the Au_22_ clusters showed a high QY emission (>60%),^[^ ^19^ ^]^ resulting from the rigidification of the Au(I)‐thiolate shell after TOA binding. Surface coverage also establishes the interfaces between the AuNP core and the outer surroundings with various biomolecules, playing important roles in biomedical applications (e.g., targeting, sensing, and imaging).^[^ ^177^, ^178^, ^179^, ^180^ ^]^ The elucidation of stimuli‐responsive surface coverage regulation from exotic targeting molecules at the ultrasmall gold surface is of great importance. Using the typical electron‐rich amines as examples, it was discovered that the nucleophilic amines could bind to electrophilic ultrasmall thiolate gold surface, which resulted in the formation of a high‐energy emission (≈600 nm) from the intrinsically low‐energy emitting (≈810 nm) AuNPs (Figure 11G–J).^[^ ^176^ ^]^ Through systematic investigation of both the structures of the exotic amine molecules and surface chemistries of the ultrasmall AuNPs, it was demonstrated that the electrophilic surface of the AuNPs provided a pathway to donate electron directly to the gold surface from the nucleophilic amines with low steric hindrance. The formed high‐energy emission as well as the decreased low‐energy emission resulted from the energy transfer between the two emissions in one single ultrasmall AuNP synergistically made the ultrasmall AuNPs robust ratiometric nanoprobes for quantitative assessment of the amine molecule interactions. The induced high‐energy emission showed an average lifetime of 0.66 µs under excitation at 405 nm, further indicating the 600 nm emission resulted from the amine interaction was involved with LMCT. Such stimuli‐responsive emission transformed from single‐emissive AuNPs to dual‐emissive ones, providing a platform to develop stimuli‐responsive ratiometric nanoprobes based on ULMNPs for quantitative bioimaging and sensing in complicated biological systems.

In addition to the biomolecules, metal ions can also stimulate the creations of new emissive centers on the surface of ultrasmall luminescent AuNPs with low surface coverage.^[^ ^181^ ^]^ Under the stimulus of trace amounts of Ag(I) ions, dual‐emissive bimetallic Ag@GS‐AuNPs with emissions at both 705 and 810 nm were formed from a single 810 nm emitting GS‐AuNP. The Ag(I) ions could interact with the Au(0) core of the 810 nm emitting GS‐AuNPs, resulting in both an antigalvanic reaction and formation of Ag(I)‐carboxylate shell on the surface of AuNPs to generate a new 705 nm emission peak in the preformed 810 nm emitting GS‐AuNPs. The dual‐emissive Ag@GS‐AuNPs with both 705 and 810 nm emissions showed unique linearly ratiometric pH‐dependent emissions. The carboxyl groups of the surface ligands were important in the formation of dual‐emissive Ag@GS‐AuNPs, and it could be adapted to other thiolate surface ligands with carboxyl groups besides GSH, which demonstrates the generality of this strategy in the formation of dual‐emissive ULMNPs toward ratiometric measurements.

## 5 Phototoxicity

Phototoxicity is a crucial consideration for live fluorescence imaging, which is mainly caused by excited fluorescent probes that can generate reactive oxygen species, heat, and DNA damage.^[^ ^182^, ^183^, ^184^ ^]^ The ULMNPs typically show fluorescence lifetimes within the microsecond time scale with triplet excited states involved in emission,^[^ ^1^ ^]^ resulting in the generation of singlet oxygen (^1^O_2_) due to the enhanced probability of energy transfer between triplet electrons from the probe and adjacent ^3^O_2_.^[^ ^185^, ^186^, ^187^, ^188^ ^]^ In disease diagnosis, the probe should generate low levels of ^1^O_2_ to reduce the side effects of phototoxicity, but disease therapy requires high efficiency in ^1^O_2_ generation.^[^ ^189^, ^190^, ^191^, ^192^ ^]^ These contradictory requirements on the level of ^1^O_2_ generation from ULMNPs naturally raise a fundamental question of how to bidirectionally regulate the ^1^O_2_ generation ability based on the different application requirements. Because many factors influence the phototoxicity (e.g., excitation wavelength, intensity, exposure time, surface chemistry of the probe, probe concentration, cell type, and age),^[^ ^193^ ^]^ we focus on the discussion on concentration and their subcellular distribution as well as the related strategies for bidirectional regulation of phototoxicity, based on the unique properties of ULMNPs.

### 5.1 Subcellular Distribution and Phototoxicity

**Figure 12:**
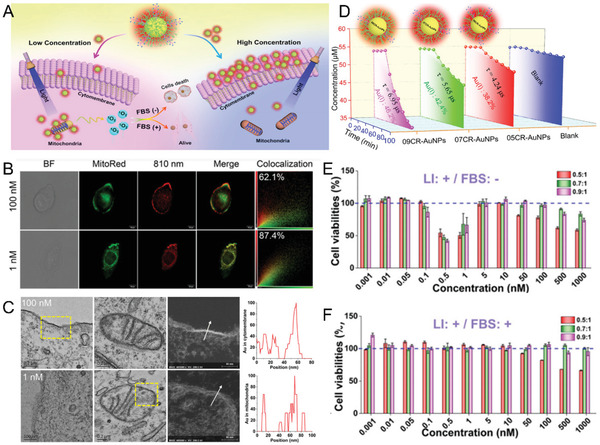
Concentration‐dependent subcellular distribution of ULMNPs and the related phototoxicity. A) Schematic diagram of concentration‐dependent subcellular distribution and ^1^O_2_ production of ULMNPs. B) Cellular imaging of AuNPs at different concentrations (100 × 10^−9^ and 1 × 10^−9^ m) and their colocalization analysis with MitoRed. Scale bar: 10 µm. C) Transmission electron microscopy (TEM), STEM images enlarged from yellow frame and EDX through white arrow of cytomembrane and mitochondria of cells incubated with 05CR‐AuNPs of 100 × 10^−9^ and 1 × 10^−9^ m. D) Generated rate of ^1^O_2_ from CR‐AuNPs at different concentrations. Cell viability of HeLa cells after incubation with CR‐AuNPs (0.001 × 10^−9^–1000 × 10^−9^ m): light illumination and FBS‐free medium (LI: +/FBS: −). E) light illumination and FBS medium (LI: + / FBS: +). F). Reproduced with permission.^[^ ^205^ ^]^ Copyright 2020, Wiley‐VCH.

The capability to control the subcellular distribution of ULMNPs not only provides unique advantages for the enhanced targeting, but also offers valid ways to fundamentally understand the cellular interaction mechanism and phototoxicity.^[^ ^194^, ^195^, ^196^, ^197^, ^198^, ^199^ ^]^ The concentration of nanomedicine significantly affects the theragnostic efficiency and systematic toxicity.^[^ ^200^, ^201^, ^202^, ^203^, ^204^ ^]^ Various nanomedicines showed concentration‐dependent biological behaviors, which resulted in different diagnostic effects and systematic toxicity at both in vitro and in vivo levels. To unveil the concentration‐dependent subcellular distribution of luminescent AuNPs, the NIR‐emitting AuNPs co‐coated with GSH and a cell‐penetrating peptide CR_8_ (CR‐AuNPs) served as versatile nanomedicines to indicate their interactions with living cells (**Figure** **12**).^[^ ^205^ ^]^ CR‐AuNPs showed strong cellular membrane‐binding capacities in high concentration regions (e.g., 500 × 10^−9^ m), whereas higher mitochondria‐targeting abilities and more relative endocytosis efficiencies were observed in the low concentration regions (e.g., 1 × 10^−9^ m). At high concentrations of CR‐AuNPs, more CR‐AuNPs were confined to the membrane, rather than endocytosis into the cell. The modification of a cell‐penetrating peptide with a local positive charge would help the CR‐AuNPs accumulate around the mitochondria at low concentrations. However, the exact mechanism for concentration dependent accumulation in mitochondria is still unclear. It would be feasible to achieve concentration‐dependent cell membrane labeling and subcellular targeting with well‐designed ULMNPs. Furthermore, this concentration‐dependent subcellular distribution resulted in photocytotoxicity for tumor cells in a peculiar low‐concentration region due to the mitochondria‐targeting generation of ^1^O_2_. These results not only demonstrate the unique concentration‐dependent biological behaviors of the ULMNPs, but also expand the understanding of ULMNPs toward future theranostics and biotoxicity.

### 5.2 Bidirectional Regulation of Phototoxicity

**Figure 13:**
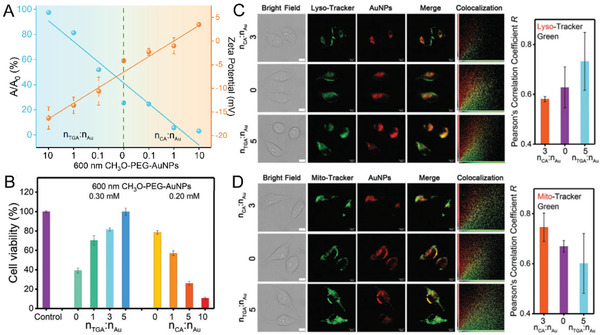
Bidirectional regulation of phototoxicity. A) The relationship between *ζ*‐potential values and the ^1^O_2_ generation rate (ABDA degradation kinetics) of AuNPs after bidirectional regulation by negatively charged thioglycolic acid (TGA) or positively charged cysteamine (CA) at different ligand to gold ratios. B) Cell viabilities of HeLa cells treated with AuNPs after regulation with TGA and CA using ligand exchange strategies at different ligand to Au ratios under light irradiation. Fluorescent images (left) and colocalization analysis (right) of AuNPs (red channel) and lyso‐tracker green C) or mito‐tracker green D) (green channel) in HeLa cells. Scale bar: 10 µm. Reproduced with permission.^[^ ^211^ ^]^ Copyright 2020, Wiley‐VCH.

For organic photosensitizers, the efficiency of ^1^O_2_ generation can be controlled by tuning the charge.^[^ ^206^, ^207^ ^]^ The increase of positively charged binding sites of organic photosensitizers favors ^3^O_2_ adsorption, which leads to the rapid and efficient formation of ^1^O_2_.^[^ ^208^, ^209^, ^210^ ^]^ This rule could also be applied to ULMNPs. It was discovered that ultrasmall PEGylated AuNPs with high surface charge showed higher ^1^O_2_ production capability than those of AuNPs with low surface charge (**Figure** **13**).^[^ ^211^ ^]^ General strategies for the controllable ^1^O_2_ production of ultrasmall AuNPs through facile surface charge regulation by thiolated ligand exchange (e.g., positively charged cysteamine; negatively charged thioglycolic acid) or deprotonation/protonation (e.g., pH regulation) have been realized. Interestingly, the surface charge of the AuNPs would affect the subcellular distributions: the introduction of negative charge would enhance the distribution of the NPs in lysosomes, but the increase in positive charge would drive the NPs to be significantly distributed in mitochondria. The cytotoxicity of the ^1^O_2_ generation was also demonstrated to be highly controllable through surface charge manipulation resulting from the synergistic effects of ^1^O_2_ generation capability and subcellular distribution. Therefore, the bidirectional regulation strategy of ^1^O_2_ generation from ultrasmall AuNPs has significant implications for the deep understanding of the photocytotoxicity of nanomedicines, as well as the future design of nanosized metal nanomedicines for specific disease diagnosis and treatment.

## 6 Conclusion and Perspective

In summary, we briefly summarize our recent progress in the surface engineering of ULMNPs for biomedical applications, such as biological targeting and imaging. Our research attempts to take advantage of the unique and fascinating surface features of ULMNPs to improve their targeting capabilities, multifunctionalities, and quantitative imaging stabilities, as well as bidirectionally regulate the toxicities based on the different application requirements. With the help of surface functionalization strategies, the ULMNPs could be designed to avoid long‐term nonspecific accumulation in MPS organs (e.g., liver and spleen) with efficient renal clearance, which not only reduces possible background interference signals in advanced imaging applications (e.g., metastatic cancer in MPS organs), but also minimize potential long‐term toxicity and systemic side effects, a prerequisite for future clinical translation. With both template‐guided and stimuli‐responsive self‐assembly strategies, the ULMNPs have been shown to be excellent platforms for developing multifunctional nanostructures in multimodal imaging (e.g., fluorescence and ultrasound) and nanomedicine (e.g., nanovectors). With the discovery of surface coverage‐regulated dual emissions from ULMNPs, ULMNPs could be easily designed as ratiometric nanoprobes without additional fluorophores for quantitative bioimaging and sensing in more complicated biological systems. The observation of unique concentration‐dependent subcellular distribution biological behaviors of the ULMNPs and the developed bidirectional regulation strategies of phototoxicity would further expand our understanding of ULMNPs toward future theranostics and biotoxicity.

Despite substantial progress made from the worldwide research groups, research on ULMNPs in biomedical application is still in its early stages, and many key issues and challenges remain unsolved. For example, the fundamental understanding of the correlation between the physicochemical properties of ULMNPs and related biological behaviors is still unclear. Because the biological behaviors of ULMNPs (e.g., cellular interaction, organ distribution, and excretion pathways) are much more sensitive to their surface chemistry (e.g., surface coverage, charge, and hydrophobicity) than those of the PMNPs, high‐quality ULMNPs with atomically precise and highly controlled surface functionalization are required to address this issue. The luminescent mechanism of ULMNPs should then be investigated in detail and systematically. Because the surface chemistry plays a significant role in governing their optical properties, a thorough investigation of the optical mechanism would sustainably develop ULMNPs with much more fascinating features (e.g., tunable optical properties and multifunctionities) for advanced bioapplications. In addition, methodologies for integration with other irradiations without the tissue penetration depth limit should be further developed. Future surface engineering strategies (e.g., self‐assembly and metal ion doping) are also needed to endow the ULMNPs with more theranostic functions, such as MRI, CT imaging, and radiotherapy. Furthermore, in addition to the cancer and renal diseases, enormous efforts should be made to explore the application of ULMNPs in other serious diseases, such as immunological, cardiovascular, and neurological diseases. Finally, the exact underlying mechanisms of physiological interactions of ULMNPs, from cells to various organs in different animal models, should be further investigated. With continued development and interdisciplinary cooperation, we believe that ULMNPs will find clinical applications in addressing many key issues related to healthcare.

## Conflict of Interest

The authors declare no conflict of interest.
